# Protective Pleiotropic Effect of Flavonoids on NAD^**+**^ Levels in Endothelial Cells Exposed to High Glucose

**DOI:** 10.1155/2015/894597

**Published:** 2015-06-09

**Authors:** Daniëlle M. P. H. J. Boesten, Saskia N. I. von Ungern-Sternberg, Gertjan J. M. den Hartog, Aalt Bast

**Affiliations:** Department of Toxicology, Maastricht University, P.O. Box 616, 6200 MD Maastricht, Netherlands

## Abstract

NAD^+^ is important for oxidative metabolism by serving as an electron transporter. Hyperglycemia decreases NAD^+^ levels by activation of the polyol pathway and by overactivation of poly(ADP-ribose)-polymerase (PARP). We examined the protective role of three structurally related flavonoids (rutin, quercetin, and flavone) during high glucose conditions in an *in vitro* model using human umbilical vein endothelial cells (HUVECs). Additionally we assessed the ability of these flavonoids to inhibit aldose reductase enzyme activity. We have previously shown that flavonoids can inhibit PARP activation. Extending these studies, we here provide evidence that flavonoids are also able to protect endothelial cells against a high glucose induced decrease in NAD^+^. In addition, we established that flavonoids are able to inhibit aldose reductase, the key enzyme in the polyol pathway. We conclude that this protective effect of flavonoids on NAD^+^ levels is a combination of the flavonoids ability to inhibit both PARP activation and aldose reductase enzyme activity. This study shows that flavonoids, by a combination of effects, maintain the redox state of the cell during hyperglycemia. This mode of action enables flavonoids to ameliorate diabetic complications.

## 1. Introduction

Worldwide more than 400 million people suffer from diabetes. This number will only grow due to the rapid increase in the incidence of the disease caused by population growth, aging, urbanization, and increasing prevalence of obesity and physical inactivity [1]. A hallmark of diabetes is hyperglycemia [2]. A number of epidemiological studies have shown a relationship between hyperglycemia and an increased risk of cardiovascular diseases, including microvascular pathologies in the eye, kidney, and peripheral nerves. As a consequence, diabetes is a leading cause of blindness, renal disease, and a variety of debilitating neuropathies (e.g., diabetic foot) [3, 4].

Nicotinamide adenine dinucleotide (NAD) is found in all living cells in an oxidized form (NAD^+^) and a reduced form (NADH). The main function of NAD in cells is modulating cellular redox status by carrying electrons from one reaction to another. Additionally, it is also involved in other cellular processes (e.g., acting as a substrate for enzymes involved in posttranslational modification) [5]. Hyperglycemia decreases NAD^+^ levels by an increased flux of glucose through the polyol pathway. This pathway becomes active when intracellular glucose levels are elevated [6]. During normoglycemia only ~3% of all glucose will enter the polyol pathway. Most of the glucose will be phosphorylated to glucose-6-phosphate by hexokinase. However, under hyperglycemic conditions ten times more glucose enters the polyol pathway [7], mainly due to a saturation of hexokinase [8]. Aldose reductase, the first and rate-limiting enzyme in the pathway, reduces glucose to sorbitol using NADPH as a cofactor. Then, sorbitol is reduced to fructose by sorbitol dehydrogenase which uses NAD^+^ as a cofactor. The osmotic stress that accompanies sorbitol accumulation and the redox imbalance following the depletion of NADPH and NAD^+^ contributes to cell damage and organ injury, ultimately leading to cataract genesis, neuropathy, and other diabetic complications [9–11].

Poly(ADP-ribose)-polymerase (PARP) activation can also lead to NAD^+^ depletion. The nuclear enzyme PARP has been implicated in the regulation of many important cellular functions like DNA repair, gene transcription, cell cycle progression, cell death, chromatin function, and genomic stability [12]. PARP detects and signals single-strand DNA breaks (SSB), which can be induced by hyperglycemia. Upon detection of a SSB, PARP binds to the DNA and synthesizes a poly(ADP-ribose) (PAR) chain as a signal for DNA repair enzymes. NAD^+^ is required as a substrate for the synthesis of these PAR monomers. Overactivation of PARP therefore depletes cellular NAD^+^ stores [13]. Several studies have suggested an important role of PARP activation in the pathogenesis of diabetic complications like nephropathy, neuropathy, and retinopathy [14–16].

Previously we have established that dietary flavonoids inhibit PARP both* in vitro* and* in vivo* [17–19]. Flavonoids are polyphenolic compounds which are found in fruits, vegetables, and plant-derived products like red wine and tea [18]. Flavonoids have been shown to display positive health effects, for example, reduced risks for cardiovascular and chronic inflammatory diseases [20–23], which have been ascribed to their antioxidant and anti-inflammatory properties [22, 24]. We now studied the effect on NAD^+^ levels in endothelial cells after exposing the cells to high glucose in the presence or absence of flavonoids. In addition we determined whether three structurally related flavonoids are also able to inhibit aldose reductase, the most important enzyme of the polyol pathway.

## 2. Material and Methods

### 2.1. Chemicals

All chemicals were purchased from Sigma-Aldrich (Steinheim, Germany) unless stated otherwise. F12K medium, Hank's balanced salt solution (HBSS), trypsin-EDTA, non-heat-inactivated fetal calf serum (FCS), and penicillin/streptomycin were obtained from Gibco (Breda, The Netherlands). Endothelial cell growth supplement (ECGS) was obtained from BD Bioscience (Breda, The Netherlands). Heparin was purchased from Leo Pharmaceuticals (Amsterdam, The Netherlands).

### 2.2. Cell Culture

Human umbilical vein endothelial cells (HUVECs) (CRL-1730) were obtained from ATCC. HUVECs were cultured in F12K medium with 10% FCS, 1% penicillin/streptomycin, 0.05 mg/mL endothelial cell growth supplement (ECGS), and 0.1 mg/mL heparin. Cells were maintained in collagen coated flasks at 37°C in a 5% CO_2_ atmosphere. For experiments, cells were seeded in 6- or 96-well plates and allowed to attach overnight. Next, medium was removed and cells were washed with HBSS. Additionally, fresh medium was added containing glucose (30 mM final concentration) or vehicle (medium) and flavonoids (5 *μ*M final concentration), sorbinil (0.5 *μ*M final concentration), or its vehicle (DMSO).

### 2.3. Gene Expression Analysis

RNA was isolated from QIAzol suspended cells according to the manufacturer's protocol and quantified spectrophotometrically with a NanoDrop. RNA (500 ng) was reverse-transcribed using iScript cDNA synthesis kit (Bio-Rad, Veenendaal, The Netherlands). Next, real time PCR was performed with a Bio-Rad MyIQ iCycler Single Color RT-PCR detection system using Sensimix Plus SYBR and Fluorescein (Quantace-Bioline, Alphen a/d Rijn, The Netherlands), 5 *μ*L diluted (10x) cDNA, and 0.3 *μ*M primers in a total volume of 25 *μ*L. PCR was conducted as follows: denaturation at 95°C for 10 minutes, followed by 40 cycles of 95°C for 15 seconds and 60°C for 45 seconds. After PCR, a melt curve (60–95°C) was produced for product identification and purity. *β*-actin was included as internal control. Primer sequences are shown in Table 1. Data were analysed using the MyIQ software system (Bio-Rad) and were expressed as relative gene expression (fold change) using the 2^ΔΔCt^ method.

### 2.4. Determination of NAD^+^ Levels

Cells were lysed with 1% dodecyltrimethylammonium bromide (DTAB) in 0.2 N NaOH. To ensure that only NAD^+^ levels were measured 0.4 M HCl was added and samples were incubated at 60°C for 15 minutes. Afterwards, cells were incubated at room temperature for 10 minutes and 0.5 M Trizma base was added to the cells after which NAD^+^ levels were determined with the NAD^+^/NADH cell based assay kit from Cayman Chemical (Ann Arbor, MI, USA).

### 2.5. Preparation of Lens Aldose Reductase

Porcine lenses were used as a source of aldose reductase enzyme. Porcine eyes were obtained from a local slaughterhouse. Lenses were removed and stored at −20°C until use. Lens homogenate was freshly prepared for every experiment. Lenses were homogenized in 1.25 mL homogenization buffer (20 mM potassium phosphate buffer, pH 7.5 containing 0.5 mM EDTA and 5 mM 2-mercaptoethanol). The homogenate was centrifuged at 10.000 ×g for 10 minutes at 4°C.

### 2.6. Aldose Reductase Assay

Aldose reductase activity was determined spectrophotometrically. The reaction mixture (0.7 mL) contained 30 mM potassium phosphate buffer (pH 6.2), 0.2 mM NADPH, 0.2 M lithium sulphate, and the substrate DL-glyceraldehyde (0–2 mM). Flavonoids (flavone, quercetin, and rutin) were added to the reaction mixture (final concentration 0.5 or 5 *μ*M). As a positive control, the known aldose reductase inhibitor sorbinil was used in a concentration of 0.5 *μ*M. Reaction was initiated by addition of NADPH. The consumption of NADPH was followed by the decrease in absorbance at 340 nm for 5 minutes at 37°C.

### 2.7. Statistical Analysis

The effect of HG incubation and effects of flavonoids were tested using Student's *t*-test for independent samples or the Mann-Whitney *U* test when not normally distributed. *P* values < 0.05 were considered statistically significant and *P* values < 0.1 were considered statistical trends. Statistical analyses were analyzed with SPSS for Windows (version 20.0; SPSS Inc., Chicago, IL, USA).

## 3. Results

**Figure 1:**
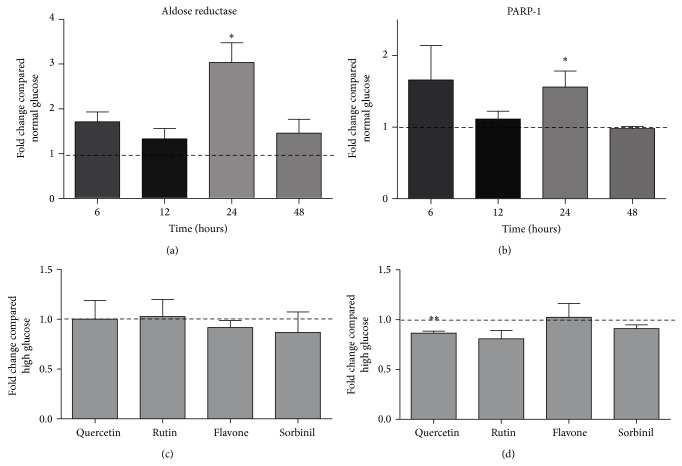
Effect of incubation with 30 mM glucose on the expression of aldose reductase (a) and PARP-1 (b) after several incubation times. Effect of addition of flavonoids (compared to high glucose incubation) after 24 hours incubation is shown in (c) (aldose reductase) and (d) (PARP-1). Data are expressed as mean ± standard error from three independent experiments. ^*∗*^ *P* < 0.05 compared to normal glucose incubation; ^*∗∗*^ *P* < 0.05 compared to incubation with high glucose alone.

In Figure 1, the effect of incubating HUVECs with 30 mM of glucose on gene expression of aldose reductase and PARP-1 is presented. It is visible that both aldose reductase and PARP-1 have a significant higher expression after 24-hour incubation compared to normal glucose. When flavonoids are coincubated during these 24 hours, there is no effect on aldose reductase expression compared to only high glucose incubation. Only quercetin seems to lower PARP-1 expression compared to high glucose incubation.

**Figure 2:**
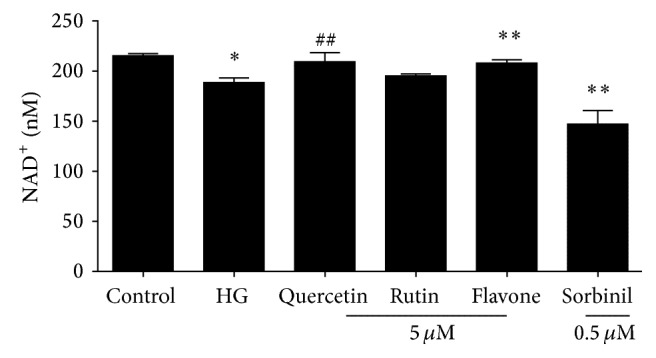
Effect of 24-hour incubation with 7 (control) or 30 mM (HG) glucose on the NAD^+^ level of HUVECs with or without coincubation with flavonoids. Data are expressed as mean ± standard error from four independent experiments. ^*∗*^ *P* < 0.05 compared to control; ^*∗∗*^ *P* < 0.05 compared to incubation with high glucose alone; ^##^ *P* < 0.1 compared to incubation with high glucose alone.

The effect of incubation with 30 mM glucose on the NAD^+^ status of HUVECs is depicted in Figure 2. High glucose incubation leads to a significant decrease in NAD^+^ levels after 24 hours. This decrease is attenuated when the cells are coincubated with flavone or quercetin (trend) but not with rutin. Incubation with the known aldose reductase inhibitor sorbinil led to an even larger decrease in NAD^+^ levels.

**Figure 3:**
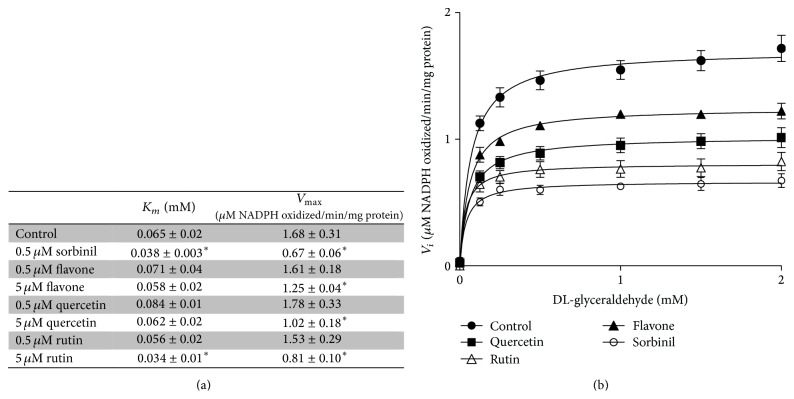
(a) Kinetics of porcine aldose reductase in the absence and presence of flavonoids. Data are expressed as mean ± standard deviation of at least three separate experiments. ^*∗*^ *P* < 0.05 compared to control. (b) An example of a Michaelis Menten plot of aldose reductase in absence (filled circles) and presence of 5 *μ*M quercetin (filled squares), 5 *μ*M rutin (open triangles), 5 *μ*M flavone (filled triangles), or 0.5 *μ*M sorbinil (open circles). Data are expressed as mean ± standard error of at least three experiments.

Quercetin, rutin, and flavone at a concentration of 5 *μ*M decreased the *V*_max_ of the aldose reductase catalysed conversion of DL-glyceraldehyde to glycerol. Sorbinil was used as a control and decreased both the *V*_max_ and *K*_*m*_ at a concentration of 0.5 *μ*M. Rutin also showed a small but significant decrease of *K*_*m*_ compared to the control (Figure 3).

## 4. Discussion

In epidemiological studies, the intake of flavonoids has been related to a reduced risk for various diseases, including diabetes [23, 25, 26]. Many complications that arise from diabetes are attributed to a redox imbalance. In previous studies we established that flavonoids were able to attenuate NAD^+^ depletion by inhibiting PARP overactivation both* in vitro* and* in vivo* [17–19]. Extending these studies, we here provide evidence that flavonoids are also able to protect endothelial cells against a decrease in NAD^+^ due to high glucose. In addition we show that flavonoids are able to inhibit the key enzyme of the polyol pathway, aldose reductase. From previous (unpublished) experiments we know that flavonoids at the concentration used in this study (5 *μ*M) do not influence glucose uptake. The flavonoids' concentration needs to be at least 10-fold higher to influence the uptake of glucose.

In this study three structurally related flavonoids were studied: flavone, the core structure of the flavonoid subgroup flavones, a compound that is present in many cereal grains as well as in dill weed [27]; quercetin, one of the most prominent dietary flavonoids present in many foods including citrus fruit and berries [28]; and rutin, a glycoside of quercetin which is found in buckwheat [29]. These compounds are usually conjugated to sugar moieties but are certainly of interest as protectors during inflammatory conditions when the pH and glucuronidase appear favorable for deconjugation as has been shown by [30].

Gene expression of aldose reductase and PARP was investigated in endothelial cells exposed to 30 mM glucose. A higher expression of aldose reductase in peripheral blood mononuclear cells has been linked to an increased risk for kidney disease in diabetic patients [31]. Furthermore, in transgenic mice, it was found that human aldose reductase expression increased atherosclerosis lesion size which could be attenuated by aldose reductase inhibitors [32, 33]. An increase in PARP mRNA expression was found in patients with type 2 diabetes and microangiopathy [34]. We found an increase in the expression of both genes when endothelial cells were exposed to 30 mM glucose for 24 hours. Coincubation with flavone, rutin, or sorbinil did not affect this increase.

NAD^+^ is a cofactor in numerous critical oxidation reactions. Because of the involvement in redox signalling, NAD^+^:NADH is regarded as one of the most important redox couples of the cells and therefore an important determinant of redox status of cells. We found a slight decrease in NAD^+^ levels after incubating HUVECs with 30 mM glucose for 24 hours. This change is most likely a combination of the two previously described pathways: a decrease in NAD^+^ due to activation of the polyol pathway and overactivation of PARP-1. Therefore we also investigated the potential of flavonoids to inhibit aldose reductase. The flavonoids' ability to inhibit aldose reductase has been described previously [35]. In our study, it was found that all tested flavonoids were able to inhibit aldose reductase enzyme activity at a concentration of 5 *μ*M. Quercetin and flavone appear to be noncompetitive inhibitors because only the *V*_max_ of the reaction is decreased. Conversely, not only did rutin decrease the *V*_max_, but it also decreased the *K*_*M*_ slightly. This would indicate a slightly higher reaction rate at very low substrate concentrations but a much lower rate at higher substrate concentrations. Rutin contains rutinose, which is a disaccharide composed of rhamnose and glucose. The latter is a substrate of aldose reductase; however the affinity of aldose reductase for DL-glyceraldehyde is higher [7]. Rutin as a competitive inhibitor is further supported by the results of sorbinil, which is a known competitive inhibitor of aldose reductase [36]. Sorbinil was tested at a lower concentration (0.5 *μ*M) but shows the same results as rutin, a decrease in both *V*_max_ and *K*_*M*_. Of the tested flavonoids, rutin showed the strongest inhibition, while flavone had the least effect. This is contrary to their capacity to inhibit PARP overactivation, where flavone is the most potent inhibitor and rutin is not able to inhibit PARP (Table 2). In both reactions quercetin is an intermediate inhibitor compared to rutin and flavone.

These findings indicate that the overactivation of PARP-1 plays a larger role than the polyol pathway in the decrease of NAD^+^ levels in HUVECs. When cells were coincubated with flavonoids, we observed that flavone was able to attenuate the decrease in NAD^+^ concentration. Flavone is the most potent PARP-1 inhibitor but did not have a large effect on aldose reductase activity. This finding is also supported by the observation that rutin, the most potent aldose reductase activity inhibitor, did not show an effect on NAD^+^ levels. Quercetin, an average inhibitor of both pathways, showed a trend towards increasing NAD^+^ levels to normal. The influence of the polyol pathway on the lower NAD^+^ level seems to be small. Most likely the activation of this pathway has a more pronounced effect on the levels of NADPH. By lowering the levels of this essential cofactor for glutathione, the cells get more susceptible to oxidative stress [37]. This in turn can lead to more reactive oxygen species that can damage DNA, inducing activation of PARP-1, which subsequently can lead to a decrease in NAD^+^ levels as we observed in HUVECs. This might also be the reason why coincubation with sorbinil leads to an extra decrease in NAD^+^ levels in HUVECs. By inhibiting the aldose reductase almost completely, unlike the flavonoids which show a mild inhibition, other pathways involved in the pathogenesis of diabetic complications may become more activated (e.g., activation PKC); this then can lead to more oxidative stress and activation of PARP-1 [37].

## 5. Conclusions

**Figure 4:**
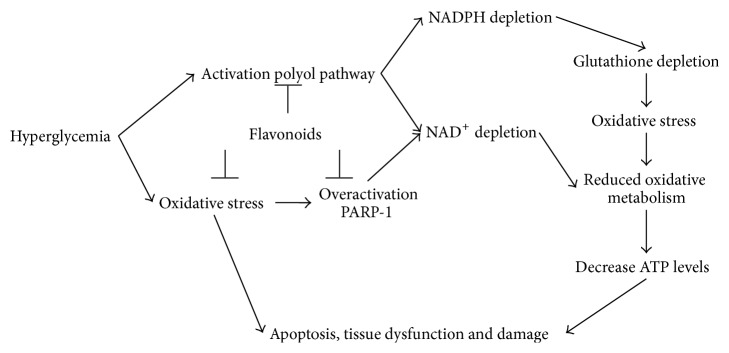
Flavonoids can protect cells under hyperglycemic stress in several ways. First, flavonoids are able to inhibit overactivation of PARP-1, preventing a decrease in NAD^+^ levels. Furthermore, flavonoids are able to inhibit aldose reductase activity, preventing an additional decrease in NAD^+^ and NADH levels. Also, because of their antioxidant properties, flavonoids are able to prevent damaging effects of oxidative stress. By a combination of all these effects flavonoids are able to protect cells against high glucose induced damage.

We conclude that flavonoids are able to exert pleiotropic protective effects under high glucose conditions (Figure 4). We observed that flavonoids were able to inhibit overactivation of PARP-1, thereby preventing a fall in NAD^+^ levels. Furthermore we observed that flavonoids are able to inhibit aldose reductase activity, preventing an additional decrease in NAD^+^ levels. Moreover, because of the known antioxidant properties of flavonoids they are also able to prevent the deleterious effects of reactive oxygen species which can be formed when a redox imbalance is present. In conclusion, the combination of all these effects is most likely the reason why flavonoids were able to protect endothelial cells against a high glucose induced drop in NAD^+^ levels in an* in vitro* system.

## Figures and Tables

**Table 1:** Primer sequences for genes used for gene expression analysis.

Gene	Forward (5′ to 3′)	Reverse (5′ to 3′)
Beta-actin (*β*-actin)	CCTGGCACCCAGCACAAT	GCCGATCCACACGGAGTACT
Aldose reductase	TACACATGGGCACAGTCGAT	GGGGTTGGGTACCTGGAA
PARP-1	GCCAGTTCAGGACCTCATCAA	CGGCCTGGATCTGCCTTT

**Table 2:** Overview of structure and PARP inhibiting capacity of flavonoids used in this study [17, 18].

Name	Flavone	Quercetin	Rutin
Structure	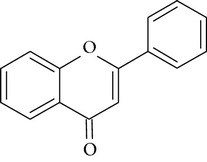	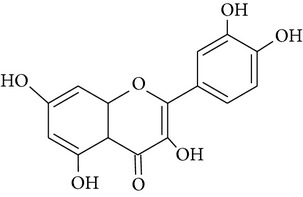	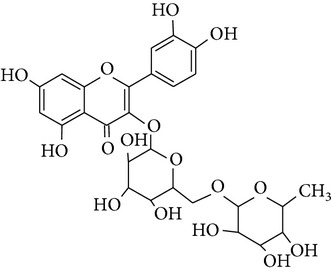
PARP inhibiting capacity	Strong, also at low concentrations	Strong, less at low concentrations	No inhibiting capacity
